# Endometrial ablation; less is more? Historical cohort study comparing long-term outcomes from two time periods and two treatment modalities for 854 women

**DOI:** 10.1371/journal.pone.0219294

**Published:** 2019-07-10

**Authors:** Liva Helleland, Lena Flekke Bergesen, Karen Jakobsen Rinnan, Ingeborg Bøe Engelsen, Knut Hordnes, Jone Trovik

**Affiliations:** 1 Department of Obstetrics and Gynecology, Haukeland University Hospital, Bergen, Norway; 2 Department of Clinical Science, University of Bergen, Bergen, Norway; 3 Department of Gynecologic outpatient care, Betanien Hospital, Bergen, Norway; National Institutes of Health, UNITED STATES

## Abstract

**Background:**

Abnormal uterine bleeding needs surgical treatment if medical therapy fails. After introduction of non-hysteroscopic endometrial ablation as alternative to hysteroscopic endometrial resection, we aimed to compare short and long-term outcomes for women treated with these two minimally-invasive procedures. A secondary goal was comparing the present cohort to a previous cohort of women treated with hysteroscopic resection only.

**Materials and methods:**

Historical cohort study of women treated for abnormal uterine bleeding with hysteroscopic resection or endometrial ablation at Haukeland University Hospital during 2006–2014. Similar patient file and patient-reported outcome data were collected from 386 hysteroscopic resections in a previous cohort (1992–1998). Categorical variables were compared by Chi-square or Fisher´s Exact-test, linear variables by Mann-Whitney U-test and time to hysterectomy by the Kaplan-Meier method.

**Results:**

During 2006–2014, 772 women were treated with endometrial resection or ablation, 468 women (61%) consented to study-inclusion; 333 women (71%) were treated with hysteroscopic resection and 135 (29%) with endometrial ablation.

Preoperative characteristics were significantly different for women treated with hysteroscopic resection compared to endometrial ablation in the 2006-2014-cohort and between the two time-cohorts regarding menopausal, sterilization and myoma status (p≤0.036). The endometrial ablation group had significantly shorter operation time, median 13 minutes (95% Confidence Interval (CI) 12–14) and a lower complication rate (2%) versus operation time, median 25 minutes (95% CI 23–26) and complication rate (13%) in the hysteroscopy group, all p ≤0.001. The patient-reported rate of satisfaction with treatment was equivalent in both groups (85%, p = 0.955). The endometrial ablation group had lower hysterectomy rate (8% vs 16%, p = 0.024). Patient-reported satisfaction rate was higher (85%) in the 2006-2014-cohort compared with the 1992-1998-cohort (73%), p<0.001.

**Conclusions:**

Endometrial ablation has similar patient satisfaction rate, but shorter operation time and lower complication rate and may be a good alternative to hysteroscopic resection for treatment of abnormal uterine bleeding.

## Introduction

Menorrhagia is a significant health problem in premenopausal women, with an estimated annual incidence for seeking medical help of 10/1000 women years [1, 2]. Menorrhagia can reduce quality of life, and cause iron deficiency anemia [3]. Treatment of menorrhagia and reversal of anemia gives an increased quality of life [4].

When specific causes of abnormal uterine bleeding such as endometrial polyps, endometrial neoplasia or hematologic bleeding disorders have been excluded, treatment may be initiated. First-line treatment has traditionally consisted of medical therapy such as progestin, either as an oral medication or as a hormone-releasing intrauterine device (IUD). However, medical therapy is often ineffective. A systematic 2016 Cochrane review revealed that 59% of women randomized to receive medical treatment for abnormal uterine bleeding underwent surgery within two years, and 77% after five years [5]. A study by Famuyide from 2017 concluded that initial radiofrequency endometrial ablation compared to medical therapy offered superior reduction in menstrual blood loss and improvement in quality of life without significant differences in total costs of care [6].

Hysterectomy is a definitive treatment of the bleeding disorder but is costly and associated with a considerable risk of severe complications [7, 8]. During the 1980s, less invasive surgical procedures were developed; removal or destruction of the endometrium while preserving the uterus.

Following the first hysteroscopic procedures, several non-hysteroscopic endometrial ablation methods have evolved during the last 25 years, including thermal balloon ablation, microwave ablation, free-fluid thermal ablation, bipolar radiofrequency ablation and cryotherapy. These newer techniques are generally quicker, have lower complication rates and are technically less demanding, allowing a more widespread use of endometrial ablation techniques [7, 9, 10].

We have earlier evaluated a large cohort of 386 patients treated with hysteroscopic resection during the time-period 1992–1998 in our department [11]. A bipolar radiofrequency endometrial ablation method was introduced as an alternative to hysteroscopic resection in our department in 2004. When altering treatment regimes, and specifically when introducing a new surgical modality, it is important to evaluate if this change is beneficial.

Hence, the objectives of this study were to evaluate the outcomes of two different minimally invasive techniques for treating abnormal menstrual bleeding in our department in the period 2006–2014, and to compare the results with a previous cohort of hysteroscopic resections only.

## Materials and methods

In this historical cohort study patients were identified from the electronic patient files from Department of Obstetrics and Gynecology, Haukeland University Hospital, Bergen, Norway. We included all patients with a Nordic Classification of Surgical Procedures (NCSP) code LCB 25 (Hysteroscopic excision of lesion), LCB28 (Hysteroscopic endometrial excision) or LCA16 (Endometrial destruction) treated during the period 2006–2014 [12]. Regarding procedure LCB25, excisions performed on polyps or uterine septa were excluded. The surgical indication for the vast majority of patients was menorrhagia unresponsive to medical treatment. According to the department guidelines, the uterine cavity length should be less than 12 cm and submucosal fibroids should be less than 5 cm, evaluated by preoperative transvaginal ultrasound. Submucosal fibroid distorting the cavity was an exclusion criterion for endometrial ablation. Endometrial samples were collected from all patients prior to surgery to exclude neoplasia. In general, no specific medical pre-treatment regimen was used.

All procedures were performed under general anesthesia or spinal block. The endometrial ablation procedures also included a paracervical nerve block. The endometrial resections were performed using a rigid 9 mm hysteroscope, bipolar loop diathermy and isotonic (0.9%) saline. The endometrial ablation procedures were performed using NovaSure® Endometrial Ablation System, (Hologic, Marlborough, Massachusetts, USA) a 6 mm diameter probe using bipolar radiofrequency diathermy for vaporization and coagulation.

We reviewed hospital records for patient information. Preoperative data included age, parity, if sterilization had been performed, preoperative medical treatment, symptoms, and ultrasound findings. Perioperative data included type of procedure, operation time and complications. Any repeat procedure or subsequent hysterectomy was noted. Between two and nine years after surgery, patients were mailed a questionnaire (S1 Fig and S2 Fig) with questions regarding short-term (<one month post-operative) and long-term (>one month post-operative) effects of treatment. Patient self-report of overall satisfaction rate finalized the questionnaire.

For peri- and postoperative analysis, the procedures were categorized into two groups: hysteroscopic endometrial resection with or without resection of fibroids and bipolar radiofrequency endometrial ablation. In the logistic regression assessing risk of subsequent hysterectomy, cases with only myoma resection performed were excluded, as this primarily is a fertility sparing treatment as opposed to full endometrial resection/ablation.

Events registered as complications were: discontinued procedure, uterine perforation, fluid deficiency >1000ml and bleeding (defined as >500ml or bleeding with the need of additional hemostatic treatment such as internal uterine compression by catheter balloon or intravenous antifibrinolytic medication).

We classified surgeons in three categories; as experienced (>50 procedures), medium experienced (10–50 procedures), or inexperienced (<10 procedures).

For comparison, and evaluation of our present treatment algorithm, we used data from a previous study of women treated with endometrial resection during the period 1992–1998 [11]. Data collection (from patient files and patient questionnaire), inclusion criteria and definitions have been exactly the same for both cohorts.

Statistical analyses were performed using the statistical package in SPSS version 25, IBM, Armonk, New York, USA. Categorical variables were compared by Chi-square test or Fisher exact test. Linear variables were compared by non-parametrical tests, Mann-Whitney U-test or Kruskal-Wallis test. Survival analysis regarding time until reoperation/hysterectomy by the Kaplan-Meier log rank method were applied to compare the two different operative procedures and to compare the two time cohorts. Logistic regression assessing risk of later hysterectomy and patient reported satisfaction was performed, evaluating preoperative clinical factors significantly different between the two surgical methods or between the two time cohorts. Factors identified as significant in univariate regression analysis were included in the multivariate model. Statistical significance is defined as p<0.05 and all tests are two-sided.

The regional ethical board (REK 2015/892) has approved this study and all women have given written consent to participation. The study is being presented according to the STROBE guidelines [13].

## Results

**Fig 1:**
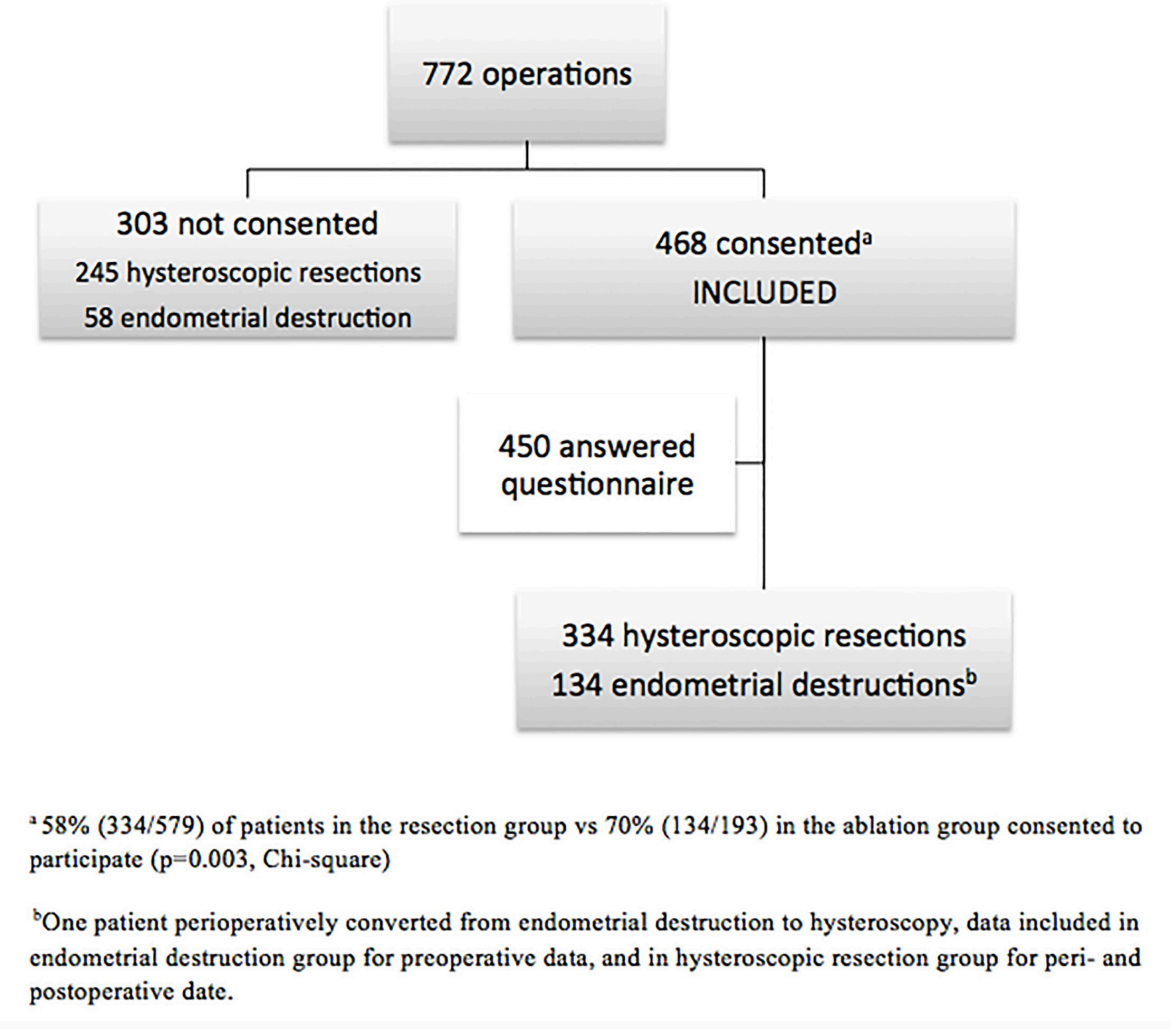
Inclusion and exclusion flowchart. Inclusion and exclusion flowchart for women treated for abnormal uterine bleeding with minimally invasive surgery at Haukeland University hospital during 2006–2014.

During the study period, 2006–2014 a total of 772 women were surgically treated with endometrial resection (75%) or endometrial ablation (25%) (Fig 1). A total of 468 patients were included after consent. Of these 450 (96%) answered the questionnaire. The procedures included were 312 (67%) hysteroscopic resections of the endometrium (with or without resection of fibroids), 21 (4%) hysteroscopic resections of fibroids only, and 135 (29%) endometrial ablations.

Preoperative clinical characteristics are shown in Table 1.

**Table 1:** Preoperative clinical characteristics for women treated for abnormal menstrual bleeding. Comparison of two minimal invasive surgical methods performed at Haukeland University hospital during 2006–2014.

	**Hysteroscopic resection n = 333** **median (95% CI**^a^**)**	**Endometrial ablation n = 135** **median (95% CI)**	**Mann-Whitney U test p-value**
Age	44 (44–45)	44 (42–45)	*0*.*079*
Parity^b^	2 (2–2)	3 (2–3)	*0*.*072*
Bleeding days per month^c^	10 (8–14)	10 (7–12)	*0*.*759*
Anteroposterior diameter uterus^d^ (mm)	48 (47–49)	48 (46–50)	*0*.*835*
Transverse diameter uterus^e^ (mm)	60 (58–62)	60 (57–64)	*0*.*920*
Endometrial height^f^ (mm)	9 (8–10)	8 (6–8)	*0*.*024*
Myoma diameter^g^ (mm)	21 (20–23)	21 (11–40)	*0*.*493*
Cavity length^h^ (cm)	9 (9–9)	9 (9–9)	*0*.*222*
	**Number (%)**	**Number (%)**	**Chi-square p-value**
Menopausal status			*<0*.*001*
Pre	291 (87)	132 (97)	
Post	42 (13)	3 (3)	
Myoma^i^			*<0*.*001*
Yes	126 (55)	14 (18)	
No	103 (45)	62 (82)	
Submucous myoma^j^			*0*.*149*
Yes	52 (41)	3 (21)	
No	74 (59)	11 (79)	
Dysmenorrhoea^i^			*0*.*052*
Yes	64 (30)	18 (19)	
No	149 (70)	75 (81)	
Previous sterilization^k^			*0*.*036*
Yes	54 (20)	34 (30)	
No	216 (80)	79 (70)	

^a^ Confidence Interval

Cases missing

^b^n = 10

^c^n = 263

^d^n = 167

^e^n = 182

^f^n = 127

^g^n = 367

^h^n = 75

^i^n = 163

^j^Out of 140 cases noted as having any myoma n = number

^k^n = 85

The 303 women declining to participate were not different from the consenting women regarding age (median 44 years, 95% CI 43–46, p = 0.753 Mann-Whitney). However, more women treated by endometrial ablation (70%) consented to participate, compared with those treated with hysteroscopic resection (58%, Fig 1).

Women treated with hysteroscopic fibroid resection only (n = 21) were younger, with a median age of 40 years (95% CI 34–44) and with a lower median parity of 0.5 (95% CI 0–1, both p ≤0.003, Kruskal-Wallis test) than women treated with hysteroscopic endometrial resection or endometrial ablation. In our further statistical analysis, the resections of fibroids only are grouped together with the endometrial resections.

Women treated with endometrial ablation had a slightly thinner endometrium measured by preoperative ultrasound and a lower rate of previous sterilization procedure than the hysteroscopic resection group. In spite of similar median age in the two groups, women treated with endometrial resection had a significantly higher proportion of postmenopausal status than in the endometrial ablation group.

Previous medical treatment to correct menorrhagia was reported in 310 (66%) of cases. Medical treatment consisted of fibrinolytic inhibitors 158/310 (34%) and/or a hormone-releasing intrauterine device (IUD) 148 (32%). Of postmenopausal women, 17/45 (38%) used any hormonal replacement therapy.

Perioperative findings comparing the two minimally invasive treatments are shown in Table 2. In total, there were significantly more complications in the hysteroscopic resection group. Overall complication rate was significantly higher if fibroids were present: 16% versus 8% (p = 0.031 Chi-square test, data not shown).

**Table 2:** Perioperative data for women treated with hysteroscopic resection or endometrial ablation during 2006–2014.

	Hysteroscopic resection n = 334 median (95% CI^b^)	Endometrial ablation n = 134^a^ median (95% CI)	Mann-Whitney U-test p-value
Operation time^c^ (minutes)	25 (23–26)	13 (12–15)	<0.001
Blood loss^d^ (ml)	50 (50–50) Range: 0–1000	0 (0–10) Range: 0–5	<0.001
	**Number (%)**	**Number (%)**	**Fisher´s exact test, p-value**
General anesthesia	317 (95)	133 (99)	0.117
Uterine perforation	14 (4)	0 (0)	0.013
Blood loss >500ml or need of treatment^e^	23 (7)	0 (0)	0.002
Fluid deficit >1000ml	14 (4)	0	0.013
Technical failure^f^	0	2	
Total perioperative complication rate	43 (13)	2 (2)	<0.001

^a^One woman scheduled for endometrial ablation converted to hysteroscopic resection due to technical problems, thus 334 completed endometrial resections and 134 endometrial ablations are used for follow-up data.

^b^CI = confidence interval

cases missing

^c^n = 7

^d^n = 316

^e^ Defined as complication if surgeon initiated measures (such as intrauterine balloon tamponade or intravenous fibrinolytic medications) to control excessive bleeding

^f^Discontinued procedure, 1 converted to hysteroscopic resection, 1 had a successfully completed ablation later same day.

n = number

In total 39 different surgeons performed the procedures during the 2006–2014 cohort. Four of the surgeons were classified as experienced and performed 273 (58%) of the procedures, of which 60% were hysteroscopies. Medium experienced surgeons performed 133 (28%) procedures and the inexperienced performed 62 (13%) procedures, of which 46 (74%) were endometrial ablations. The surgeons classified as inexperienced performed only 16 (5%) of the hysteroscopic resections. There were no statistical significant differences in complication rates among the different groups of surgeons, (p = 0.073).

Histopathologic examination of the hysteroscopic resectate detected malignancy in 2 (0.04%) women, one despite benign preoperative histological sample and one where preoperative endometrial sample was missing. Both women had a hysterectomy performed during the first postoperative month.

The short-term and long-term postoperative variables were not significantly different when comparing the two treatments (Table 3).

**Table 3:** Patient self-reported clinical characteristics after surgery for abnormal uterine bleeding during 2006–2014.

Short-term post-operative (≤1 month)	Hysteroscopic resection n = 334^a^ median (95% CI^b^)	Endometrial ablation n = 134 median (95% CI)	Mann-Whitney U test, p-value
Pain (days)	2 (1–3)	2 (2–3)	0.200
Bleeding (days)	6 (5–7)	7 (4–7)	0.635
Sick leave (days)	4 (3–7)	7 (3–7)	0.225
**Long term post-operative** **(>1 month)** **n = 450 answered questionnaire**	**n (%)**	**n (%)**	**Chi-square test, p-value**
Satisfied^c^			0.955
Yes	270 (85)	112 (85)	
No	49 (15)	20 (15)	
Bleeding^d^			0.459
Reduced	227 (85)	101 (88)	
Unchanged	29 (11)	6 (5)	
Augmented	11 (4)	8 (7)	
Amenorrhoic			0.646
Yes	130 (42)	54 (41)	
No	179 (58)	77 (59)	
Pain change dysmenorrhea group^e^			0.520
Reduced/unchanged	23 (62)	8 (73)	
Augmented	14 (38)	3 (28)	
Pain change overall^f^			0.959
Reduced/unchanged	137 (77)	63 (77)	
Augmented	42 (23)	19 (23)	
Subsequent hysterectomy (n = 468)			0.024
Yes	54 (16)	11 (8)	
No	280 (84)	123 (92)	

^a^One woman scheduled for endometrial ablation converted to hysteroscopic resection due to technical problems, thus 334 completed endometrial resections and 134 endometrial ablations are used for follow-up data.

^b^CI = confidence interval

Cases missing

^c^n = 17

^d^n = 86

^e^n = 34

^f^n = 208, n = number

Only 20 women (4%) reported persistent bleeding disorder in the questionnaire, 15 after hysteroscopic resection and 5 after endometrial ablation (p = 0.647 Chi-square test). We found no significant difference in pain change amongst the endometrial resection and the endometrial ablation groups, irrespective of pre-existing dysmenorrhea.

**Fig 2:**
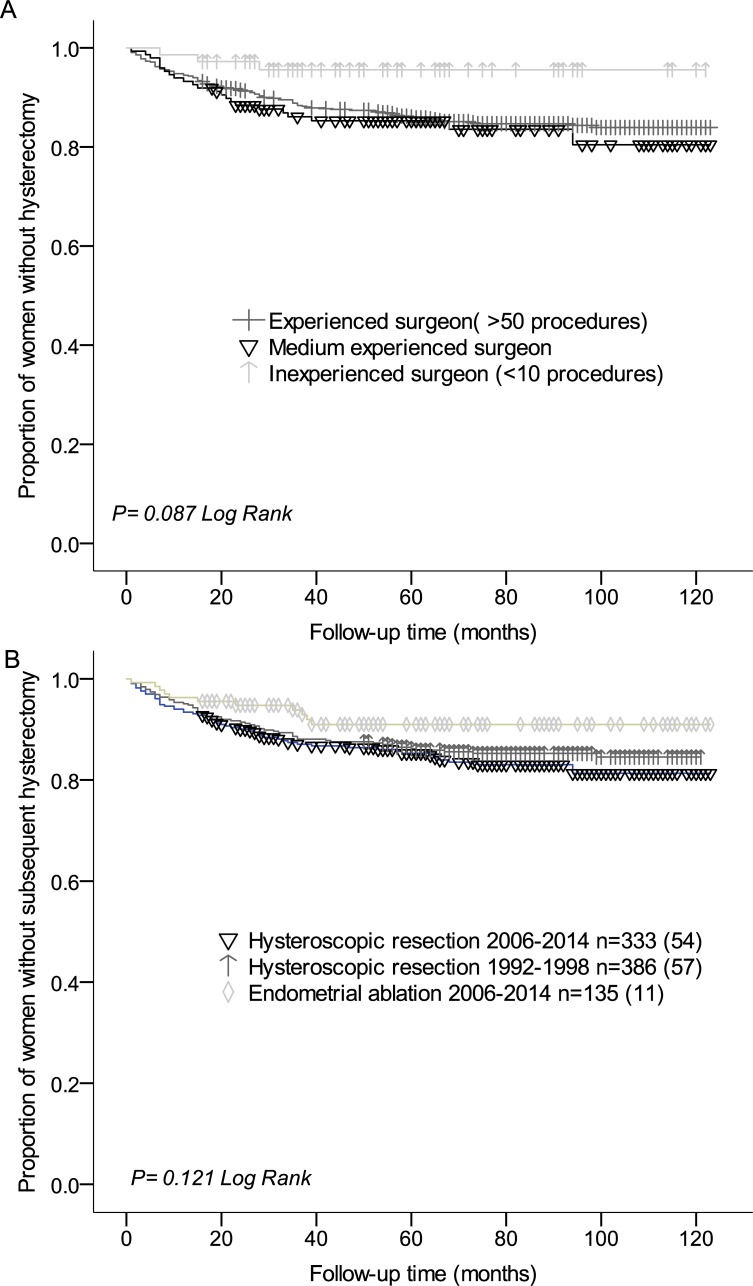
Survival curves displaying rate of subsequent hysterectomy. Rate of subsequent hysterectomy after hysteroscopic resection or endometrial ablation for 843 women treated during 1992–1998 and 2006–2014. A. Grouped based on surgeon experience level. B. Grouped based on time-cohort and surgical technique.

The median follow-up time for the 2006-2014-cohort was 76 months (Fig 2A). During this period, 54 (16%) women in the hysteroscopic resection group had a subsequent hysterectomy performed, compared with 11 (8%) women in the endometrial ablation group (p = 0.024). The time until hysterectomy was median 15 months (95% CI 10–28) in the endometrial ablation group, and 19 months in the hysteroscopy group (95% CI 6–38, p = 0.726). The proportion of patients reporting satisfaction with the received treatment was identical for both treatments. Surgeon experience did not significantly influence the rate of hysterectomy.

Regarding the preoperative clinical characteristics, the two time-cohorts differ only in a significantly higher proportion of postmenopausal women and more myomas described preoperatively while fewer women were subjected to sterilization in the present cohort (2006–2014) (Table 4).

**Table 4:** Preoperative clinical characteristics for women treated for abnormal menstrual bleeding during two time periods. Women treated by hysteroscopic resection (n = 334) and endometrial ablation (n = 135) at Haukeland University hospital during 2006–2014, compared to a cohort treated by hysteroscopic resection only during 1992–1998.

	**2006–2014** **n = 468** **median (95% CI**^a^**)**	**1992–1998** **n = 386** **median (95% CI)**	**Mann-Whitney U test p-value**
Age	44 (43–45)	44 (43–45)	*0*.*269*
Parity^b^	2 (2–3)	3 (2–3)	*0*.*103*
Bleeding days per month^c^	10 (8–12)	9 (8–10)	*0*.*078*
Endometrial height^d^ (mm)	9 (8–9)	8 (8–9)	*0*.*459*
Cavity length^e^ (cm)	9 (9–9)	9 (9–9)	*0*.*272*
	**Number (%)**	**Number (%)**	**Chi-square p-value**
Menopausal status			*<0*.*001*
Pre	423 (90)	380 (98)	
Post	45 (10)	6 (2)	
Myoma^f^			*0*.*018*
Yes	140 (46)	190 (55)	
No	165 (54)	154 (45)	
Submucous myoma^g^			*<0*.*001*
Yes	55 (39)		
No	85 (61)		
Dysmenorrhoea^h^			*0*.*444*
Yes	100 (33)	97 (30)	
No	207 (67)	229 (70)	
Previous sterilization^i^			*<0*.*001*
Yes	88 (23)	181 (64)	
No	295 (77)	101 (36)	

^a^Confidence Interval

Cases missing

^b^n = 18

^c^n = 338

^d^n = 282

^e^n = 118

^f^n = 205

^g^of n = 333 women with documented myoma n = number

^h^n = 222

^i^n = 190

Comparing perioperative data from the two different time-cohorts, the procedures during 2006–2014 were more often performed in general anesthesia rather than regional anesthesia, had a shorter operation time and a lower complication rate (Table 5).

**Table 5:** Perioperative data for women treated by minimal invasive procedures during two time cohorts. Women treated by hysteroscopic resection (n = 334) or endometrial ablation (n = 134) during 2006–2014, compared with hysteroscopic resection during 1992–1998.

	2006–2014 n = 468 median (95% CI^a^)	1992–1998 n = 386 median (95% CI)	Mann-Whitney U-test p-value
Operation time^b^ (minutes)	20 (19–22)	31 (30–35)	<0.001
	**Number (%)**	**Number (%)**	**Fisher´s exact test, p-value**
General anesthesia	450 (96)	126 (33)	<0.001
Uterine perforation	14 (3)	31 (8)	0.001
Blood loss >500ml or need of intervention^c^	23 (5)	25 (6)	0.890
Fluid deficit >1000ml	14 (3)	22 (6)	0.049
Total procedures with any major complication	45 (10)	69 (18)	<0.001

^a^CI = confidence interval

cases missing

^b^n = 15

^c^Defined as complication if surgeon initiated measures (such as intrauterine balloon tamponade or intravenous fibrinolytic medications) to control excessive bleeding, n = number

The questionnaire used to evaluate outcomes were answered by 96% of included patients in our present cohort. The outcomes regarding early post-operative bleeding, pain and rate of subsequent hysterectomy were similar when comparing the two time-cohorts (Table 6 and Fig 2B). However, a higher proportion of women in the present cohort reported to be satisfied with the procedure and more women became amenorrhoic, compared with the previous cohort.

**Table 6:** Patient self-reported clinical characteristics after surgery for abnormal uterine bleeding, comparison of two cohorts.

Short-term post-operative (≤1 month)	2006–2014 n = 468 median (95% CI^a^)	1992–1998 n = 386 median (95% CI)	Mann-Whitney U-test p-value
Pain^b^ (days)	2 (2–2)	2 (0–2)	0.247
Bleeding^c^ (days)	6 (5–7)	6 (5–7)	0.781
Sick leave^d^ (days)	5 (3–7)	7 (7–7)	<0.001
**Long term post-operative** **(>1 month)** n = 450 answered questionnaire	**n (%)**	**n (%)**	**Chi-square test, p-value**
Satisfied^e^			<0.001
Yes	382 (85)	157 (73)	
No	69 (15)	57 (27)	
Amenorrhoic^f^			<0.001
Yes	184 (42)	57 (27)	
No	258 (58)	157 (73)	
Persisting bleeding problem^g^			0.161
Yes	20 (4)	6 (2)	
No	422 (96)	243 (98)	
Subsequent hysterectomy			0.715
Yes	65 (14)	57 (15)	
No	403 (86)	329 (85)	

^a^CI = confidence interval

Cases missing: ^a^n = 365

^b^n = 312

^c^n = 299

^d^n =

^e^n = 190

^f^n = 198

^g^n = 164

n = numbers

When assessing the risk of subsequent hysterectomy after endometrial resection or ablation we excluded cases with only myoma resection performed. Neither endometrial height, menopausal status, presence of myomas nor former sterilization were of individual (univariate) statistical significance. When adjusting for potential confounders (age at treatment and surgeon experience), neither the new treatment modality (endometrial ablation) nor patient being treated during the present cohort had any increased risk of subsequent hysterectomy (Table 7).

**Table 7:** Prediction of risk of subsequent hysterectomy after minimal invasion surgery for bleeding disorders. Uni- and multivariate logistic regression for 823 patients treated at Haukeland University Hospital 1992–1998 and 2006–2014.

Variable	n	Univariate OR	95% CI	*p*-value	Multivariate OR	95% CI	*p*-value
Age at treatment (years)	823	0.95	0.92–0.98	0.003	0.95	0.92–0.98	0.001
Cohort treated				0.581			0.389
1992–1998	375	1			1		
2006–2014	448	0.90	0.61–01.3		1.20	0.79–1.82	
Type surgery				0.025			0.103
Endometrial ablation	135	1			1		
Hysteroscopic resection	688	2.10	1.1–4.02		1.8	0.89–3.70	
Surgeon experience				0.004			0.027
Inexperienced	71	1			1		
Moderate/Experience	252	6.36	1.54–26.29		5.19	1.21–22.35	

n: number of patients, OR: odds ratio

Assessing patient self-reported satisfaction only former sterilization was of statistical significance when testing in univariate analysis. Neither age, surgeon experience nor complication rates were significantly different. The rate of patients reporting to be satisfied with their surgery was borderline higher in the 2006–2014 cohort with an OR of 1.77 (95% CI 1.03–3.03, p = 0.040) adjusted for type surgery and sterilization status (S1 Table). Type surgery was not independently a factor influencing patient satisfaction.

## Discussion

Comparing the two time cohorts including 854 women treated with minimally invasive procedures for abnormal uterine bleeding, we found a stable subsequent hysterectomy rate and a slightly improved patient-reported satisfaction rate. Comparing the two minimally invasive procedures, the endometrial ablation, despite being performed by less experienced surgeons, was found to have fewer complications but a similar rate of patient-reported satisfaction and a non-inferior subsequent hysterectomy rate compared with treatment by hysteroscopic resection.

When introducing a new treatment modality it should lead to a benefit for the patients with improved efficacy and/or reduced complication rate without increased use of resources.

A large Cochrane review from 2019, including 28 trials with a total of 4287 women, demonstrated that endometrial ablation techniques (“second generation endometrial ablation”) resulted in a similar improvement of abnormal uterine bleeding as hysteroscopic endometrial ablation (“first generation endometrial ablation”) when measuring pictorial blood assessment charts (PBAC)[8]. They reported an amenorrhea rate of 39% for both ablative techniques. We found a 42% amenorrhea rate in our present cohort, no significant difference between endometrial resection and endometrial ablation, but a significant improvement from the 27% amenorrhea rate reported in our previous cohort. In the same review [8], the total patient-reported satisfaction rate was equal at 91% for second generation and 90% for first generation ablation techniques. We found an 85% satisfaction rate for both modalities in the present cohort, showing a significant increase from 73% in our previous cohort.

The complication rates have been significantly reduced in the present cohort compared with our previous cohort. Still, the overall hysteroscopic complication rate of 13% is substantial compared with the rate of 0.81% found by Jansen in 2000 [14]. However these rates are not directly comparable. In order not to underestimate complication rates, in both our cohorts, we registered all procedures with a set volume of blood loss or fluid deficit as complications, while the Jansen-study defined an event as complication only when leading to intervention. Perforation was noted in 8% of the hysteroscopies in our previous cohort and 4% in the present cohort, the latter approaches the 1% risk noted in a study from 2007 of 600 procedures [15]. This study reported experienced surgeons only in contrast to only 58% of experienced surgeons in our present cohort. The Cochrane review from 2019 states a perforation rate of 1.3% for hysteroscopic resection and 0.4% for endometrial ablation. The significantly reduced overall complication rate in our present cohort can probably be related to the introduction of the less technically challenging and less risky radiofrequency endometrial ablation technique.

We found a 12 minutes significantly shorter operation time in the endometrial ablation group compared with the hysteroscopic resection groups. This is supported by the 2019 Cochrane review reporting a median reduction of 13.5 minutes when using endometrial ablation techniques [8]. We also acknowledge that the hysteroscopic procedure requires more preoperative theatre time for instrument set-up, while the radiofrequency ablation instrument is more easily prepared. Unfortunately, we had not recorded data of the total theatre time in our two cohorts, and cannot estimate the impact of this assumed added benefit.

Subsequent hysterectomy rate of 16% for a hysteroscopic resection compared with 8% for endometrial ablation is in favor of the latter. Similar rates are reported in a Finnish long-term follow-up after endometrial ablation and in a study of endometrial ablation for patients diagnosed with adenomyosis, both with a subsequent hysterectomy rate of 19% [16, 17]. Similar pattern of difference was also found in the 2019 Cochrane review regarding hysterectomy at 2-5years; 19% after hysteroscopy versus 16% after endometrial ablation [8].

However, in our logistic regression, accounting for potential confounders, we did not find endometrial ablation to independently neither increase nor decrease the risk of subsequent hysterectomy as compared to hysteroscopic resection.

We found no significant difference in reduced pain between the hysteroscopic resection and the endometrial ablation group, irrespective of pre-existing dysmenorrhea. This supports a study of radiofrequency ablation treatment for adenomyosis, noting a significant decrease in dysmenorrhea rate in 47% of their patients [17]. Also, this finding challenges a tradition of not choosing endometrial ablation for patients with preoperative substantial dysmenorrhea. We acknowledge that as fibroids distorting the cavity was an exclusion criteria only for endometrial ablation, and the complication rate for hysteroscopic resection when fibroids are present are higher, some of the difference in outcome and satisfaction rates might be accounted for by this difference in patient population. However, our patient files are not standardized regarding description of myomas and their relation to the uterine cavity, thus we did not exclude patients from our hysteroscopic resection group based on this criterion. However the rate of reported submucous myoma was not significant different between the groups subjected to hysteroscopic resection versus endometrial ablation. Still the hysteroscopy group and the cohort operated 2006–2014 were noted with higher rates of myomas, in the univariate logistic regression neither myomas in total or submucosal location was identified of significance in affecting rate of neither subsequent hysterectomy nor patient satisfaction.

Our two time-cohorts are largely comparable, but differ in some aspects. The present cohort has a significantly higher proportion of postmenopausal women, 45 (10%) versus six (2%) in the previous cohort. Except for contributing to a generally higher rate of achieving amenorrhea in the present cohort, we do not see this difference as an important confounder. Tested in logistic regression it was not of statistical significance in relation to hysterectomy rate nor patient satisfaction.

Surprisingly, we found that 2% of women treated with endometrial ablation were postmenopausal, not in accordance with our department guidelines. This treatment modality is not intended for postmenopausal women, and might be a potentially dangerous intervention when it comes to evaluation for malignancy.

In the present cohort, 19% of the women had a prior sterilization procedure, in contrast to 50% in our previous cohort. This difference is probably due to a substantial increase in patient cost for sterilization procedures, introduced in Norway in 2002. From approximately 20 Euro (NOK 193) before 2002 to 635 Euro (NOK 6079) after, leading to a decline in female sterilization rate by more than 70% [18]. Although women with a former sterilization reported higher patient satisfaction in univariate regression analysis this factor was no longer significant in the multivariate model. We therefore do not consider the reduction of sterilization as an important confounder regarding outcomes from the minimally invasive procedures.

In two women, histopathologic examination of the resectate detected malignancy. The risk of endometrial cancer has been estimated as 0.33% for women with premenopausal abnormal bleeding [19] and 9% for women with postmenopausal bleeding [20]. With a considerable higher risk of endometrial cancer amongst women with postmenopausal bleeding, we consider hysteroscopic procedure yielding histologic tissue for histopathologic examination as the preferred modality for these women. It has been disputed whether hysteroscopy may cause intraperitoneal spread of malignant cells through the fallopian tubes, but this has been refuted [21]. The risk of finding malignant cells in hysteroscopic material calls for extra attention to have fresh endometrial biopsies preoperatively and to choose endometrial ablation with caution when treating women long time after menopause.

The main limitation of this study is that it is a non-randomized, retrospective single-center study. Furthermore, we lack PBAC-scores for objective evaluation of the bleeding patterns, but we include patient-reported outcomes regarding change in bleeding. The overall response rate in this newest cohort was 60%, which is significantly lower than the 85% participation rate in our former study cohort. Women treated by hysteroscopic resection had a significantly lower response rate than those treated by endometrial ablation. Whether this influence the patient reported treatment satisfaction rate we do not know. If those less satisfied opt out of answering, this may mask a difference in patient reported satisfaction between the different procedures. Similarly if those opting out have a higher rate of failure (need of further treatment) this may also mask a reported difference between the two methods, although for the participating women we do not find any difference regarding satisfaction or subsequent hysterectomy.

We have tried to evaluate the impact of relevant confounding factors: age, menopausal status, sterilization status, endometrial thickness, presence of myomas and surgeon experience by performing multivariate logistic regression analysis. Of these factors only higher age independently decreased risk of subsequent hysterectomy while women treated by surgeons of moderate to high experience somewhat surprisingly had a higher rate of further surgery. We assume this is related to more “complex” patients (as noted with higher rates of myomas and slightly thicker endometrium) were allocated to hysteroscopic resection by experienced surgeons while junior doctors using endometrial ablation treated the less complicated patients.

The strength of our study is the relatively large patient cohorts regarding the total number of hysteroscopic resections and endometrial ablations, and a long follow-up including patient reported outcomes performed similarly in two different time-periods. Surgeons were not specifically assigned to participate in this study when performing the procedures. Therefore, our findings reflect a success- and complication rate to be expected in everyday surgical practice. Our study includes outcome in relation to surgeon experience and highlights the fact that less surgical experience has no impact on complication or satisfaction rate for non-hysteroscopic endometrial ablation. Our hospital is a tertiary hospital serving 10% of the Norwegian population, and we assume that our cohort is representative for the average Norwegian female population. The validity of our study is considered to extend at least to the other Nordic countries, as the populations and the quality of health services are similar. All together, we consider that our findings are clinically meaningful and may have direct implication on surgical practice. Thus, complementing hysteroscopic treatment for abnormal bleeding by an endometrial ablation procedure has been evaluated as beneficial in a routine clinical setting.

## Conclusions

After introduction of endometrial ablation as a supplement to hysteroscopic resection, the complication rate and operation time in our department has declined, compared with a previous cohort of hysteroscopic resection. Although demanding less surgeon experience, the patient reported outcomes were slightly better and the subsequent hysterectomy rate non-inferior for patients operated during 2006–2014 when endometrial ablation was part of routine treatment for abnormal uterine bleeding. When medical treatment is not feasible, endometrial ablation may be a good alternative to hysteroscopic resection in properly selected patients.

## Supporting information

S1 Fig Patient-reported questionnaire, Norwegian. Norwegian version (original) of questionnaire used.
(DOCX)

S2 Fig Patient-reported questionnaire, English. English translation of questionnaire used.
(DOCX)

S1 Table Prediction of patient self-reporting overall satisfaction with minimal invasion surgery for bleeding disorders. Uni- and multivariate logistic regression for 508 patients treated at Haukeland University Hospital 1992–1998 and 2006–2014.
(DOCX)
