# Supplementary material for: Endometrial ablation; less is more? Historical cohort study comparing long-term outcomes from two time periods and two treatment modalities for 854 women
Source: PLoS One. 2019 Jul 10;14(7):e0219294. doi: 10.1371/journal.pone.0219294 (PMC6619760; doi:10.1371/journal.pone.0219294)
Supplement: S1 Fig — Norwegian version (original) of questionnaire used. (DOCX) [file pone.0219294.s001.docx]

**Spørreskjema**

**Behandling av blødningsplager ved fjerning av livmorslimhinnen: Evaluering av behandling i to tidsperioder**

| **Den første tiden etter inngrepet (ca. første måneder):** | |
| --- | --- |
| Hvor lenge blødde du (dager)? |  |
| Hvor lenge var du sykmeldt (uker)? |  |
| Hvor lenge hadde du smerter?  Var du fornøyd med inngrepet: Ja Nei Vet ikke |  |
|  |  |
| **Senere:** |  |
| Har du hatt blødning senere: Ja Nei |  |
| Hvis ja, hvor mange dager pr måned: |  |
| Er du kommet i overgangsalderen (menopause) etter inngrepet: Ja Nei Vet ikke |  |
| Hvis ja: Hvilket år stoppet menstruasjonen endelig: |  |
| Er blødningen nå mindre økt eller uforandret i forhold til før inngrepet? |  |
| Dersom du var bra en tid etter inngrepet og siden ble verre - hvor lenge var du bra: |  |
| Er smertene ved menstruasjon nå mindre økt eller uforandret i forhold til før inngrepet? |  |
| Har du brukt prevensjon etter inngrepet? Ja Nei |  |
| Har du vært gravid etter inngrepet? Ja Nei |  |
| Har du brukt hormontabletter eller hormonplaster etter inngrepet Ja Nei |  |
| Har du måttet ha noen ny operasjon mot blødningene Ja Nei  Hvis ja: hvilken typer operasjon: Og når ble du operert igjen: (årstall) |  |
|  |  |
|  |  |
| **Viktigst:** |  |
| Oppfatter du blødningene som et problem fortsatt Ja Nei |  |

| **Kommentarer:** |  |
| --- | --- |
