# Supplementary material for: Endometrial ablation; less is more? Historical cohort study comparing long-term outcomes from two time periods and two treatment modalities for 854 women
Source: PLoS One. 2019 Jul 10;14(7):e0219294. doi: 10.1371/journal.pone.0219294 (PMC6619760; doi:10.1371/journal.pone.0219294)
Supplement: S2 Fig — English translation of questionnaire used. (DOCX) [file pone.0219294.s002.docx]

**Questionnaire**

**Treatment of bleeding disorders by removal of the endometrial lining: Evaluation of treatment during two time cohorts.**

| **The time early following treatment (First few months):** | |
| --- | --- |
| For how long after surgery did you experience bleeding (days)? |  |
| For how long did you receive sick-leave (weeks)? |  |
| For how long did you experience pain?  Were you satisfied with the surgery: Yes No Unsure |  |
|  |  |
| **Later:** |  |
| Have you experienced bleeding later: Yes No |  |
| If yes; how many days per month: |  |
| Have you entered menopause after the surgery: Yes No Unsure |  |
| If yes: Which year did your periods finally stop: |  |
| Is your bleeding decreased increased or unchanged compared to before the surgery? |  |
| If you felt better a while after surgery but later got worse, for how long were you well: |  |
| Are any menstrual pains increased decreased or unchanged after the surgery? |  |
| Have you used contraception after surgery? Yes No |  |
| Have you been pregnant after the surgery? Yes No |  |
| Have you used hormone tablets or patches after the surgery? Yes No |  |
| Have you received any additional surgery for your bleeding problems ? Yes No  If yes: which type of surgery: When was this new surgery: (year) |  |
|  |  |
|  |  |
| **Most important:** |  |
| Do you consider bleeding a present problem? Yes No |  |

| **Comments:** |  |
| --- | --- |
