# Supplementary material for: Endometrial ablation; less is more? Historical cohort study comparing long-term outcomes from two time periods and two treatment modalities for 854 women
Source: PLoS One. 2019 Jul 10;14(7):e0219294. doi: 10.1371/journal.pone.0219294 (PMC6619760; doi:10.1371/journal.pone.0219294)
Supplement: S1 Table — Uni- and multivariate logistic regression for 508 patients treated at Haukeland University Hospital 1992–1998 and 2006–2014. (DOCX) [file pone.0219294.s003.docx]

S1 table. **Prediction of patient self reporting overall satisfaction with minimal invasion surgery for bleeding disorders**. Uni- and multivariate logistic regression for 508 patients treated at Haukeland University Hospital 1992-1998 and 2006-2014.

| Variable | n | Univariate OR | 95% CI | *p*-value | Multivariate OR | 95% CI | *p*-value |
| --- | --- | --- | --- | --- | --- | --- | --- |
| Cohort treated  1992-1998  2006-2014 | 157  351 | 1  2.33 | 1.46-3.69 | <0.001 | 1  1.77 | 1.03-3.03 | 0.040 |
| Former sterilization  No  Yes | 323185 | 1  1.99 | 1.26-3.15 | 0.003 | 1  1.58 | 0.96-2.61 | 0.072 |
| Type surgery  Endometrial ablation  Hysteroscopic resection | 111397 | 1  0.53 | 0.29-1.00 | 0.051 | 1  0.72 | 0.37-1.43 | 0.348 |

n: number of patients, OR: odds ratio
